# Coral reef aorta, emergency surgical: case report and literature review

**DOI:** 10.1590/S1679-45082014RC2772

**Published:** 2014

**Authors:** Sergio Quilici Belczak, Igor Rafael Sincos, Ricardo Aun, Kaline Viana Costa, Etianne Andrade Araujo

**Affiliations:** 1 Hospital Geral de Carapicuíba, Carapicuíba, SP, Brazil; 2 Hospital Israelita Albert Einstein, São Paulo, SP, Brazil

**Keywords:** Aorta, Aorta, abdominal, Atherosclerosis, Endarterectomy, Ischemia, Emergencies, Case reports

## Abstract

Coral reef aorta is described as an uncommon entity characterized by the presence of coarse calcifications, which are developed in the visceral aorta. These calcifications grow toward the lumen of the artery and can result in significant stenosis, so that causing malperfusion of the lower limbs, visceral ischemia or hypertension secondary to renal involvement. We report here a case of a 54-year-old patient with coral reef aorta and symptomatic. The clinical presentation of the patient required the surgical approach. A review of literature in major databases was conducted to compare health-related aspects of the disease presentation and management. Coral reef aorta should be considered as the diagnosis for patients with visceral and limbs ischemia. The approach in our case was consistent with other studies previously published in the literature.

## RESUMO

A aorta em recife de corais é descrita como uma entidade incomum caracterizada pela presença de calcificações grosseiras, que se desenvolvem na aorta visceral. Essas calcificações crescem em direção ao lúmen da artéria e podem resultar em estenose significativa, gerando má perfusão dos membros inferiores, isquemia visceral ou hipertensão secundária ao acometimento renal. Relata-se aqui o caso de um paciente de 54 anos portador de aorta em recife e sintomático. O quadro clínico do paciente exigiu abordagem cirúrgica. Foi realizada revisão de literatura nas principais bases de dados para comparar os aspectos relacionados à apresentação e à conduta da doença. Em pacientes com sinais de isquemia visceral ou em membros inferiores, deve-se considerar o diagnóstico de aorta em recife de coral. A abordagem, no caso relatado, foi consistente com estudos publicados anteriormente na literatura.

## INTRODUCTION

Coral reef aorta (CRA) is a rare disease characterized by atherosclerosis, calcification, and stenosis of the abdominal aorta, being described as rock-hard calcifications in the visceral part of the aorta. These calcifications often resemble the growths of hyperplastic bone, though abnormalities in serum calcium are not found. These heavily calcified plaques grow into the lumen and can cause significant stenosis, which may lead to malperfusion of the lower limbs, visceral ischemia or hypertension due to renal ischemia.^(1)^

**Figure 1:**
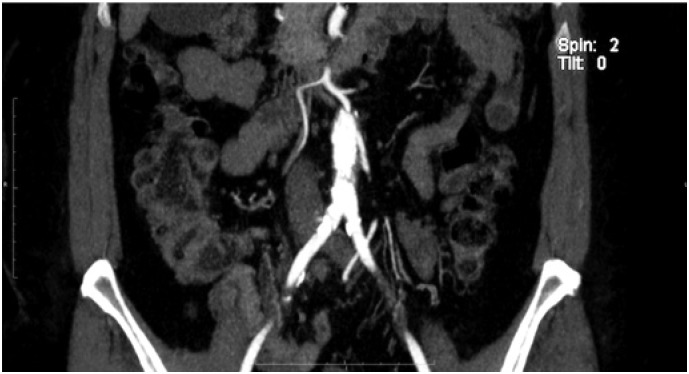
Angiotomography showing aortic atherosclerotic lesion obstructing the aorta

This disease entity was first described in 1984, when Qvarfordt et al. published their findings of a series of nine patients with obstructive lesions of the suprarenal aorta, which they named “coral reef aorta” because the lesions resembled those oceanic structures (Figure 1).^(2,3)^ It is estimated that fewer than 200 cases of CRA have been reported in the literature worldwide since 1984. For this reason, a more in-depth look at this condition is still lacking.

The symptoms and clinical presentation of CRA depend on the disease extent and involved aortic branches.^(4)^ The most common preoperative symptoms are severe hypertension and intermittent claudication,^(5)^ although other clinical presentations have already been reported.^(4, 6–10)^

The diagnosis is based on unique aspects of this condition, including internal appearance of the calcified plaques within the vessel and endoluminal protruding calcifications more extensive than those observed in routine atherosclerosis. The location of lesions is a valuable diagnostic information, because CRA tends to be seen primarily in the juxtarenal and suprarenal locations.^(11)^

CRA lesions tend to be located on the posterior surface of the aortic wall. Computed tomography (CT) angiography or arteriography of the entire abdominal aorta in the anterolateral direction should be performed for diagnostic confirmation. In addition, the intraluminal thrombus should be considered in the differential diagnosis.^(1,11)^ CT angiography plays an important role in identifying the extent of calcifications and defining the optimal site of aortic clamping for surgical management.^(11)^

Differential diagnosis includes the “shaggy aorta” syndrome and the middle aortic syndrome (MAS). “Shaggy aorta” syndrome is a rare disease associated with both ulcerated, thrombus-lined lesions and clinically evident, repeated embolic episodes. Differently from CRA, vessel wall lesion is predominantly represented by thrombus rather than calcifications at image assessment.^(12)^ MAS affects abdominal aorta in children and young adults. The main difference regarding CRA is the involvement of the visceral branches of the aorta. Segmental aortic stenosis may be located at the suprarenal, inter-renal or infrarenal aorta, with a high propensity for concomitant stenosis in both the renal (63%) and visceral (33%) arteries.^(13)^

Although some authors reported patients with CRA who did not undergo surgical treatment and remained asymptomatic throughout the follow-up period, the majority of authors consider CRA potentially life-threatening if left untreated, because it may lead to renal and visceral ischemia.^(1)^

The treatment of choice depends on the aspect of the lesion. For open repair, transaortic thromboendarterectomy can be carried out with a longitudinal incision into the affected segment of the aorta and the removal of calcified plaque. When the lesion extends substantially along the visceral aorta or the renal arteries, bypass is the open procedure that should be performed.^(1,14)^ When the quality of the affected segment cannot be ensured after the procedure, an open graft replacement is required.^(1)^ In some cases, the lesion site, and renal and visceral vessel involvement preclude the balloon angioplasty or stent placement. In cases of favorable anatomy, the primary choice is the endovascular treatment, which can be successfully performed with stent implantation.^(15)^

The appearance of dissected aortic specimens has been described as resemble a coral reef, with extensive calcifications that form an intraluminal mass. Optimally, histological examination should be performed only after a long period of decalcification. The Van Gieson's staining shows hyalinized parietal fibrin layers grouped into coral reef-like structures. The intima is composed of several layers of collagenous and elastic fibers. Both the intima and the media exhibit dystrophic calcifications, and some specimens may show evidence of early-stage heterotopic ossification. Hemosiderin deposition and foam cells have been found in some patients.^(6)^

This report derived from a case of CRA. The patient was managed and followed-up at the vascular surgery service of a private hospital in the city of São Paulo (SP, Brazil). A literature review was carried out in an attempt to gather and present useful information on the optimal management of this condition and to cover aspects such as the etiology, pathogenesis, clinical presentation, diagnosis and treatment of CRA.

## CASE REPORT

A 54-year-old woman was admitted to our service with a 6-month history of intermittent claudication with sudden and progressive worsening in the days before her admission and with bilateral cyanosis of the toes. She reported severe lower extremity pain (10, pain scale zero to 10). She had no history of diabetes, hypertension, dyslipidemia, other comorbidities nor surgical history of note, but she was a longstanding smoker.

Physical examination revealed bilateral absence of femoral and distal pulses, and a trophic ulcer on the right foot, with partial necrosis of the hallux. Laboratory exams were normal. There was no evidence of renal impairment.

**Figure 2:**
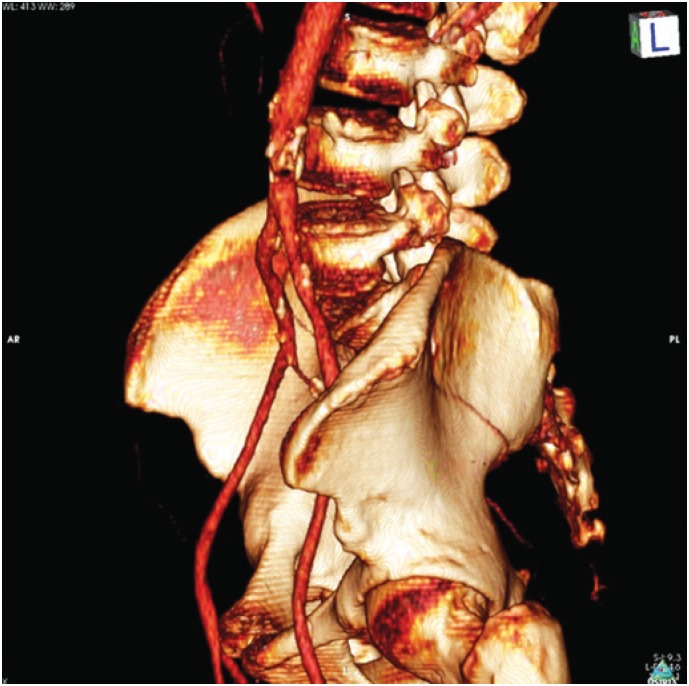
Angiotomography with a tridimensional reconstruction showing aortic atherosclerotic lesion obstructing the aorta

**Figure 3:**
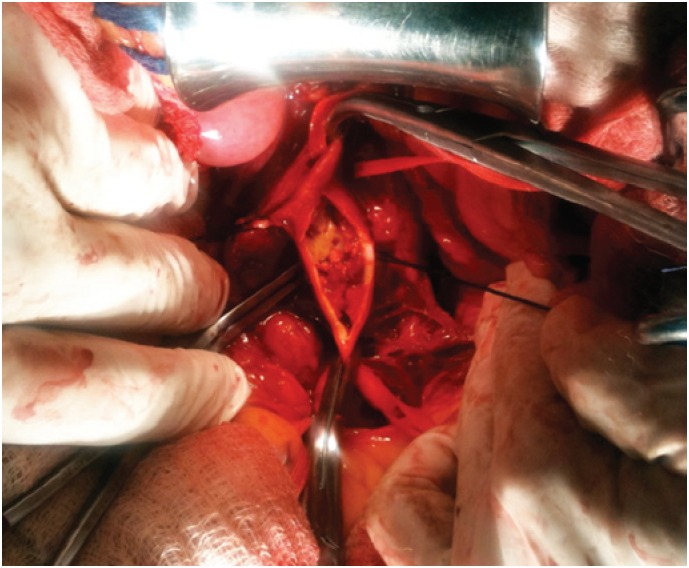
Intraoperative image showing the atherosclerotic plaque in the aorta

**Figure 4:**
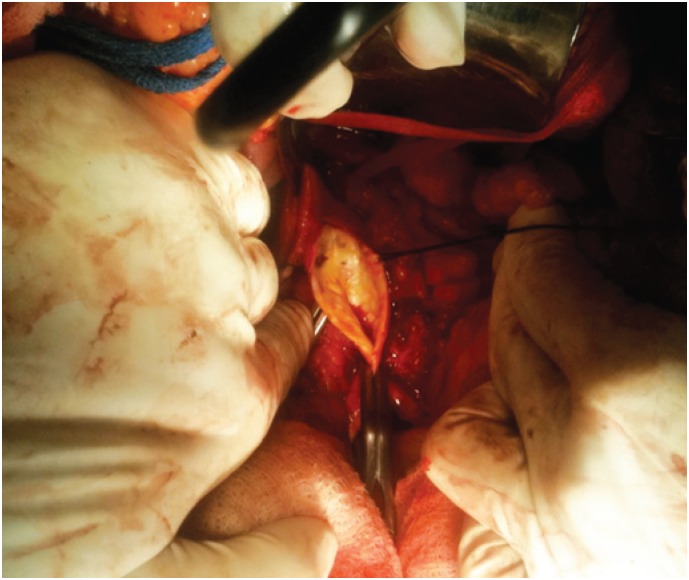
Intraoperative image showing the aorta with complete removal of atheromatous plaque

**Figure 5:**
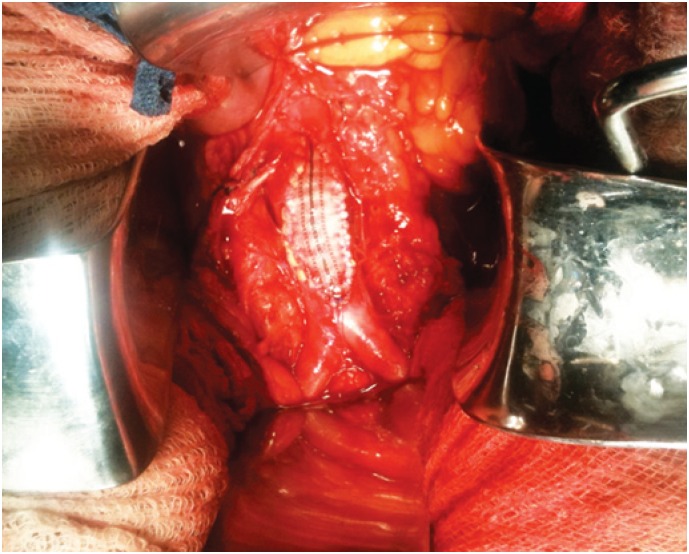
Intraoperative image showing the aorta closed with a patch of Dacron

Angiotomography revealed an irregular, extensive, subtotal occlusive lesion of the infrarenal aorta (Figures 1 and 2). The patient underwent urgent aortic endarterectomy with Dacron patch angioplasty (Figure 3). Atheromatous plaque was completely removed and also the complete reperfusion of the affected extremities was achieved (Figures 4 and 5). Currently, the patient is asymptomatic and has returned to daily activities. Her trophic lesions are fully healed.

## DISCUSSION

The search in PubMed/MEDLINE retrieved 24 articles on CRA. A total of 17 articles were selected because of their clear and objective approach to the topic.

Reports on calcification and obstruction of the abdominal aorta were rare before the 1980s. In 1984, Qvarfordt et al. described a series of nine patients with isolated stenosis of the suprarenal aorta due to coarse calcifications.^(3)^ Since then, many authors have been reporting case series of patients diagnosed with similar lesions. Unlike the first report, the majority of later studies have not found any significant difference in gender distribution of patients with CRA. Those individuals affected are usually 40 to 50 years old.^(4,6)^

Schulte et al. reported a series of 21 patients and the most common symptoms were hypertension (17 patients), intermittent claudication (13 patients), abdominal pain (7 patients), impaired renal function (6 patients), lower extremity pain at rest (1 patient), and end-stage renal disease (1 patient). All patients underwent vascular surgical procedures, including open thromboendarterectomy of suprarenal (43%), infrarenal (19%), or supra-and infrarenal aorta (38%), and also thromboendarterectomy of the following vessels: celiac artery (33%), superior mesenteric artery (57%), inferior mesenteric artery (14%), unilateral renal artery (14%), or bilateral renal artery (43%). Bypass reconstructions were performed in 39% of cases.^(9)^

In a series of 70 patients with CRA reported by Grotemeyer et al., the most frequent finding was renovascular hypertension, which caused vertigo, headaches, and visual symptoms in 44.3% of patients. All patients had developed severe stenosis of the renal artery or suprarenal aorta due to protrusion of the coral reef like calcifications. Intermittent claudication due to peripheral arterial occlusive disease was found in 27 patients (38.6%). The pain-free walking distance was less than 200m (Fontaine stage IIb) in 20 patients (74.1%). Fifteen patients had chronic visceral ischemia that caused diarrhea, weight loss and abdominal pain. Abdominal angina occurred in 11 of the 15 patients (73.3%), and weight loss in 12 of them (80.0%). Sixty-nine patients (98.6%) underwent surgery; in 57 patients, aortic reconstruction was achieved with thromboendarterectomy performed on an isolated suprarenal segment (8.7%), infrarenal segment (21.7%), and the supra- and infrarenal aorta (62.3%).^(4)^

In a study by Sagban et al. with 80 patients, the most common finding was renovascular hypertension (n=33; 41.3%) that caused headache, vertigo, and visual symptoms. Intermittent claudication due to peripheral arterial occlusive disease was reported in 35 patients (43.8%). Fifteen (19%) patients had chronic visceral ischemia, which led to diarrhea, weight loss, and abdominal pain. Of the 80 patients in the series, 79 (98.7%) underwent surgical treatment, which consisted of aortic reconstruction by thromboendarterectomy in 73 cases, isolated suprarenal segment in 7 patients, infrarenal segment in only 21 patients, and both segments in 45 patients (60%). Renal arteries and aortic bifurcation clearance was performed in 47 and 37 patients, respectively. Surgical access was by left-sided thoracoabdominal incision in 45 patients (56.4%) and by laparotomy in 33 (41.8%). The 30-day mortality rate was 8.7% (7 patients).^(7)^

Four studies on CRA were published between 2009 and 2012. One was a follow-up study of a previously published case series and the remaining three were new reports with cases similar to those reported by Sagban et al.^(7)^ (Chart 1).

**Chart 1:** Literature review

Author	Title	Patients	Findings	Management	Outcome
Sagban et al. 2010^(7)^	Occlusive aortic disease as coral reef aorta	80	Hypertension, headache, vertigo, visual symptoms, and intermittent claudication	Thromboendarterectomy	79 patients underwent surgery, 7 died
Schlieper et al. 2010^(8)^	*Analyse des calcifications chez les patients ayant une atteinte coralliforme de l'aorte*	10	Hypertension, intermittent claudication, and visceral ischemia	Open or endovascular repair	Association between arterial calcifications and low serum level of α2-HS glycoprotein (fetuin A)
Satsu et al. 2011^(9)^	Congestive heart failure due to coral reef thoracoabdominal aorta	1	Hypertension, hyperlipidemia, bowel ischemia, and congestive heart failure	Left-sided axillofemoral bypass	The study procedure was beneficial for patients with cardiac dysfunction
Sugimoto et al. 2009^(10)^	Extraanatomic bypass and coronary artery grafting for coral reef	1	Hypertension, diabetes mellitus, hypothyroidism, and abdominal pain	Extra-anatomic bypass and coronary artery grafting	Resolution of digestive symptoms and claudication

Unlike most cases previously reported in the literature, our patient presented intermittent claudication as the predominant symptom, but she did not have renovascular hypertension, which is usually a cardinal finding in CRA.

## CONCLUSION

Coral reef aorta has been the subject of relatively little research, but it is a highly relevant condition from the clinical standpoint. Some cases can be presented as a surgical emergency, thus requiring prompt diagnosis and treatment. The delayed management can result in catastrophic consequences including visceral and renal infarction.
